# Regulation of CCR7-dependent cell migration through CCR7 homodimer formation

**DOI:** 10.1038/s41598-017-09113-4

**Published:** 2017-08-17

**Authors:** Daichi Kobayashi, Masataka Endo, Hirotaka Ochi, Hironobu Hojo, Masayuki Miyasaka, Haruko Hayasaka

**Affiliations:** 1 Laboratory of Immune Regulation, Department of Microbiology and Immunology, Osaka University Graduate School of Medicine, 2-2 Yamada-oka, Suita, Osaka 565-0871 Japan; 2 Laboratory of Immune Molecular Function, Faculty of Science & Engineering, Kindai University, 3-4-1 Kowakae, Higashiosaka, Osaka 577-8502 Japan; 3 Institute for Protein Research, Osaka University, 3-2 Yamada-oka, Suita, Osaka 565-0871 Japan; 4 Interdisciplinary Program for Biomedical Sciences, Institute for Academic Initiatives, Osaka University, 2-2 Yamada-oka, Suita, Osaka 565-0871 Japan; 5 MediCity Research Laboratory, University of Turku, FIN-20520 Turku, Finland; 6 WPI Immunology Frontier Research Center, Osaka University, 2-2 Yamada-oka, Suita, Osaka 565-0871 Japan; 7 Department of Pharmacology, Wakayama Medical University, 811-1 Kimiidera, Wakayama, 641-0012 Japan

## Abstract

The chemokine receptor CCR7 contributes to various physiological and pathological processes including T cell maturation, T cell migration from the blood into secondary lymphoid tissues, and tumor cell metastasis to lymph nodes. Although a previous study suggested that the efficacy of CCR7 ligand-dependent T cell migration correlates with CCR7 homo- and heterodimer formation, the exact extent of contribution of the CCR7 dimerization remains unclear. Here, by inducing or disrupting CCR7 dimers, we demonstrated a direct contribution of CCR7 homodimerization to CCR7-dependent cell migration and signaling. Induction of stable CCR7 homodimerization resulted in enhanced CCR7-dependent cell migration and CCL19 binding, whereas induction of CXCR4/CCR7 heterodimerization did not. In contrast, dissociation of CCR7 homodimerization by a novel CCR7-derived synthetic peptide attenuated CCR7-dependent cell migration, ligand-dependent CCR7 internalization, ligand-induced actin rearrangement, and Akt and Erk signaling in CCR7-expressing cells. Our study indicates that CCR7 homodimerization critically regulates CCR7 ligand-dependent cell migration and intracellular signaling in multiple cell types.

## Introduction

Recruitment of lymphocytes from the blood into secondary lymphoid tissues is a process contributing to continuous immune surveillance. This process is tightly regulated by the interaction between lymphoid chemokines expressed in lymphoid tissues and their specific G-protein-coupled receptors in migrating cells^1, 2^. CCR7 is one of the major chemokine receptors preferentially expressed in a wide range of immune cells, including naïve T and B cells, central memory T cells, mature dendritic cells^3^, and plasmacytoid dendritic cells^4, 5^. CCR7 interacts with CCR7 ligands (CCL19 and CCL21) expressed mainly in the high endothelial venules (HEVs) and lymph node parenchyma^3^. Gene knockout mice lacking CCR7 or CCR7 ligands show marked impairment of T cell migration into lymphoid organs, indicating that CCR7 signaling is indispensable for T cell recruitment *in vivo* ^3^. Recent reports suggest that CCR7 is also involved in the progression of various diseases such as HIV-1 infection and dissemination^6, 7^, systemic lupus erythematosus (SLE)^8^, pneumonia^9^, malaria^10^, allergy^11^, and tumor metastasis to lymph nodes^12^.

A large body of evidence suggests that the majority of chemokine receptors form constitutive or ligand-induced homo- and/or heterodimers, and that these dimers modulate receptor activities^13, 14^. CCR5 dimerization is necessary for positively regulating CCR5-dependent cellular responses such as calcium flux and cell migration *in vitro* and *in vivo* ^15^. CXCR4 homodimers constitutively formed in malignant cells have been suggested to play important roles in cell migration, and ligand-induced CXCR4 dimerization activates CXCR4-mediated downstream signaling pathways^16, 17^. Recently, CCR7 homodimerization by CCL21 was reported to induce Src kinase-dependent signaling and promote efficient cell migration^18^. In addition to homodimerization, chemokine receptor heterodimerization is thought to affect chemokine-mediated signaling. Heterodimerization between CCR2 and CCR5 increases the sensitivity and range of the chemokine response^19^. CXCR7 and CXCR4 heterodimerization regulates CXCR4-mediated intracellular signaling in T cells^20^. Thus, chemokine receptor dimerization is one of the key mechanisms in chemokine receptor-mediated signaling.

In our previous report, we showed that the sensitivity of CCR7 to its ligand chemokines correlated with the levels of CCR7 homo- and CXCR4/CCR7 heterodimerization^6^. These observations support the idea that chemokine receptor homo- and/or heterodimerization play an important role, although the exact contribution of the receptor dimerization to CCR7-dependent signaling events remains unclear.

In this report, we examined the contribution of CCR7 dimerization to CCR7-dependent cell migration and signaling in detail by inducing CCR7 homo- or heterodimerization using a chemically-induced dimerization (CID) method. We showed that CCR7 homodimerization promoted CCL19-Ig fusion protein binding and CCR7 ligand-dependent T cell migration, whereas inhibition of CCR7 homodimerization by a CCR7-derived synthetic peptide attenuated CCL19-Ig binding, CCL19-induced CCR7 internalization, CCL19-induced actin rearrangement, CCR7-dependent intracellular signaling, and CCR7-dependent T cell migration. CCR7 homodimerization thus critically regulates CCR7 ligand-dependent cell migration and intracellular signaling.

## Results

### Induction of CCR7 homodimer formation enhances CCL19-Ig binding and CCR7 ligand-induced cell migration

**Figure 1:**
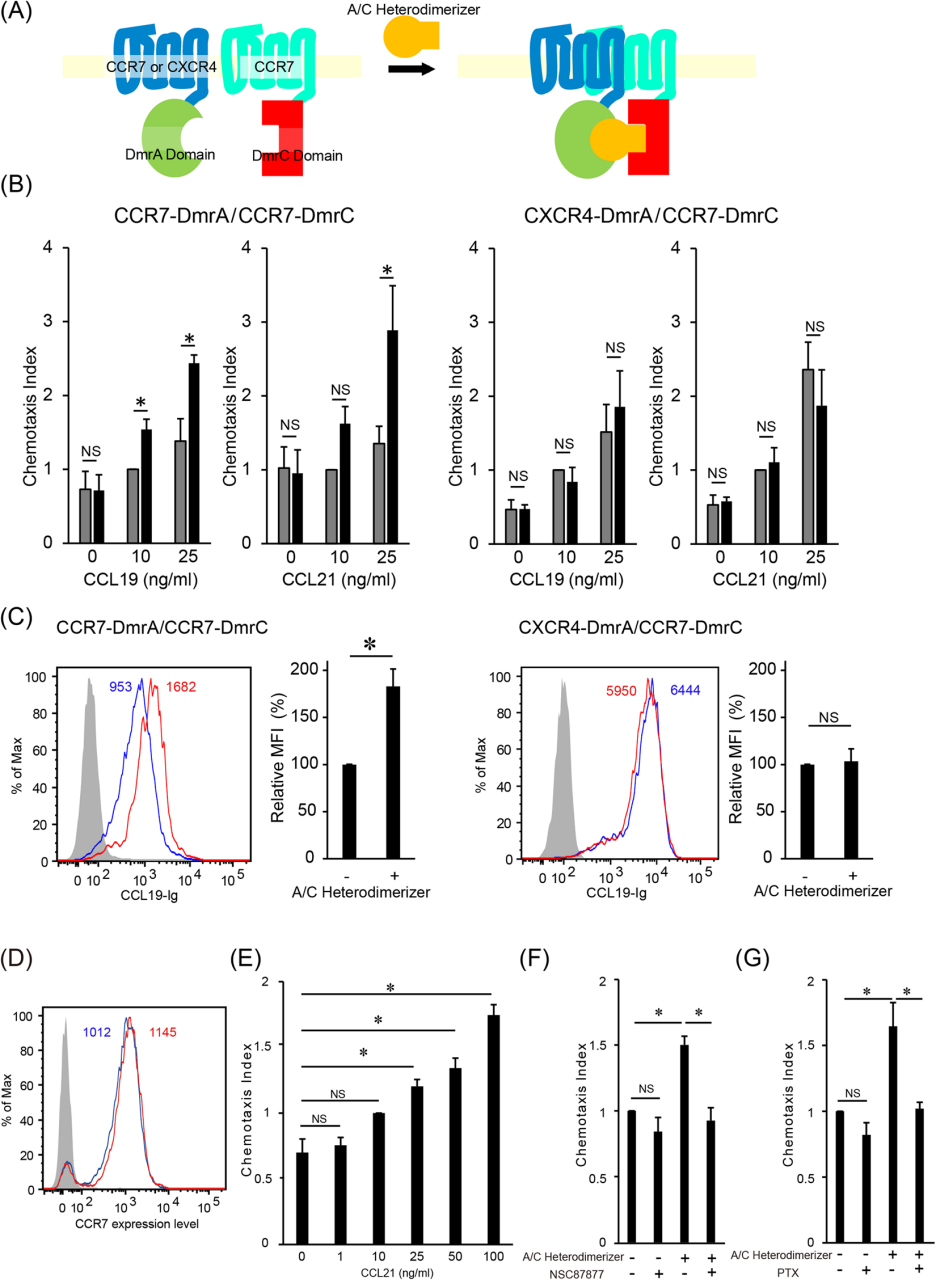
Induction of CCR7 homodimer formation enhances CCR7-dependent chemotaxis and CCL19 binding. (**A**) Schematic diagram of artificial CCR7 homo- and CXCR4/CCR7 heterodimer formation by a CID system. (**B**) CCR7-dependent chemotaxis after induction of CCR7 homo- (left) or CXCR4/CCR7 hetero (right) -dimerization was analyzed by the Transwell assay. CCL19 or CCL21 at the indicated concentration was added to the lower wells, and T cells were added to the upper wells in the presence (black bars) or absence (gray bars) of A/C Heterodimerizer. The relative chemotactic index shown is the ratio of the number of cells that migrated at the indicated concentration of CCL19 or CCL21 to those that migrated at 10 ng/ml CCL19 or CCL21 without A/C Heterodimerizer. Data represent mean ± SEM from three or four independent experiments. (**C**) Binding levels of CCL19-Ig in the presence (red) or absence (blue) of A/C Heterodimerizer. The gray-filled histogram shows the staining using isotype-matched control immunoglobulin. The median fluorescence intensity (MFI) is indicated on the histograms (left). The relative MFI of CCL19-Ig binding with or without A/C Heterodimerizer is shown (right). Data are shown as mean ± SD from three independent experiments. (**D**) CCR7 expression levels in CCR7-DmrA/CCR7-DmrC cells were examined by flow cytometry. Cells were stained with anti-human CCR7 antibody after treatment with (red) or without (blue) A/C Heterodimerizer. The gray-filled histogram represents staining with isotype control antibody. MFI is indicated on the histograms. (**E**) CCR7-DmrA/CCR7-DmrC cell migration in response to CCL21. The relative chemotactic index shown is the ratio of the number of cells that migrated at the indicated concentration of CCL21 to those that migrated at 10 ng/ml CCL21. Data represent mean ± SEM from four experiments. (**F**) The effect of NSC 87877 (40 μM, left) or PTX (100 ng/ml, right) on CCR7-DmrA/CCR7-DmrC cell migration after induction of CCR7 homodimerization. The relative chemotactic index achieved by the number of migrated cells in response to 25 ng/ml CCL21 is shown. Data represent mean ± SEM of three (NSC87877) and six (PTX) experiments. *p < 0.05 by Student’s *t* test; NS, not significant.

To assess the contribution of CCR7 homodimer formation and CXCR4/CCR7 heterodimer formation to CCR7-dependent cell migration, we artificially manipulated the level of CCR7 homo- or CXCR4/CCR7 heterodimerization in human T cells, using a CID system (Fig. 1A)^21^. We first established the H9 human T cell line lacking the CXCR4 and CCR7 genes using the CRISPR/Cas9 system to exclude the contributions of endogenous CXCR4 and CCR7, and then generated H9 cells stably expressing either DmrA-tagged CCR7 or DmrA-tagged CXCR4 together with DmrC-tagged CCR7 (Supplemental Fig. 1). In these cells, a proportion of CCR7 spontaneously formed homodimers and heterodimers, respectively (Supplemental Fig. 2A and B). We subsequently induced homodimerization of CCR7 molecules by adding a high-affinity ligand for the Dmr domains, A/C Heterodimerizer, to the CCR7-DmrA/CCR7-DmrC expressing H9 cells. As expected, the CCR7 homodimer level was increased by this treatment (Supplemental Fig. 2A), whereas it was unaffected in the parental H9 cells under these conditions (Supplemental Fig. 3). The level of CXCR4/CCR7 heterodimer was also increased by A/C Heterodimerizer treatment in the CXCR4-DmrA/CCR7-DmrC expressing cells (Supplemental Fig. 1B). The A/C Heterodimerizer-treated cells were then subjected to CCR7-dependent cell migration assay at different concentrations of CCL19 and CCL21 (Fig. 1B and Supplemental Fig. 4). When CCR7 homodimerization was induced, the cells showed a substantial increase in cell migration at low concentrations of CCL19 (10 and 25 ng/ml) (Fig. 1B, left panel), but not at higher concentrations (50 and 100 ng/ml) (Supplemental Fig. 4, left panel). Comparable results were achieved with CCL21 (Fig. 1B, left panel, Supplemental Fig. 4, left panel), where the cells showed an increase in cell migration at low concentrations (25 and 50 ng/ml), but not at a higher concentration (100 ng/ml). Induction of CCR7 homodimerization did not affect CCR1-dependent cell migration (Supplemental Fig. 5), indicating that the enhancing effect of CCR7 homodimerization was selectively observed in the cells responding to CCR7 ligand chemokines. In contrast, induction of CXCR4/CCR7 heterodimerization did not affect cell migration at any ligand concentration tested (Fig. 1B, right panel, Supplemental Fig. 4, right panel). From these results, we hypothesized that CCR7 homodimerization contributes to the enhanced cell migration at low CCR7 ligand concentration more significantly than heterodimerization.

We next examined the effect of CCR7 homodimerization or CXCR4/CCR7 heterodimerization on the level of CCL19-Ig fusion protein binding. Induction of CCR7 homodimerization increased the level of CCL19-Ig binding by approximately two-fold in CCR7-DmrA/CCR7-DmrC cells (Fig. 1C, left panel) without affecting the CCR7 expression level on the cell surface (Fig. 1D). Although the basal CCL19-Ig binding level was higher in the cells co-transfected with CXCR4-DmrA and CCR7-DmrC than those expressing only CCR7, induction of CXCR4/CCR7 heterodimerization did not further affect CCL19-Ig binding (Fig. 1C, right panel). These results support the hypothesis that CCR7 homodimerization but not CXCR4/CCR7 heterodimerization enhances CCR7 signaling by increasing CCR7 ligand binding in T cells.

A recent report suggests that a tyrosine phosphatase SHP2 is activated by CCR7 dimerization and is critical for CCL21-induced signaling and migration^18^. We thus assessed the involvement of SHP2 in the enhanced cell migration observed subsequent to CCR7 homodimerization. At a minimum effective CCL21 concentration (25 ng/ml, Fig. 1E), the enhancing effect by A/C Heterodimerizer was significantly restored by an SHP1/2 inhibitor, NSC 87877, to the level without A/C Heterodimerizer (Fig. 1F, left panel). Another SHP2 inhibitor, PHPS1, also suppressed the effect of A/C Heterodimerizer comparably (Supplemental Fig. 6A). In the absence of A/C Heterodimerizer, neither NSC87877 nor PHPS1 significantly affected cell migration (Supplemental Fig. 6B). These results imply that CCR7 homodimerization acts upstream of SHP2 signaling to mediate CCL21-induced cell migration. We next examined involvement of Gαi signaling in the enhanced cell migration by CCR7 homodimerization. As shown in Fig. 1F right panel, CCL21-induced cell migration in the presence of A/C Heterodimerizer was significantly reduced by pertussis toxin (PTX) to the level without A/C Heterodimerizer, suggesting that Gαi signaling is involved in the enhanced cell migration.

### CCR7 homodimers are polarized toward leading edge during CCR7 ligand-dependent cell migration

**Figure 2:**
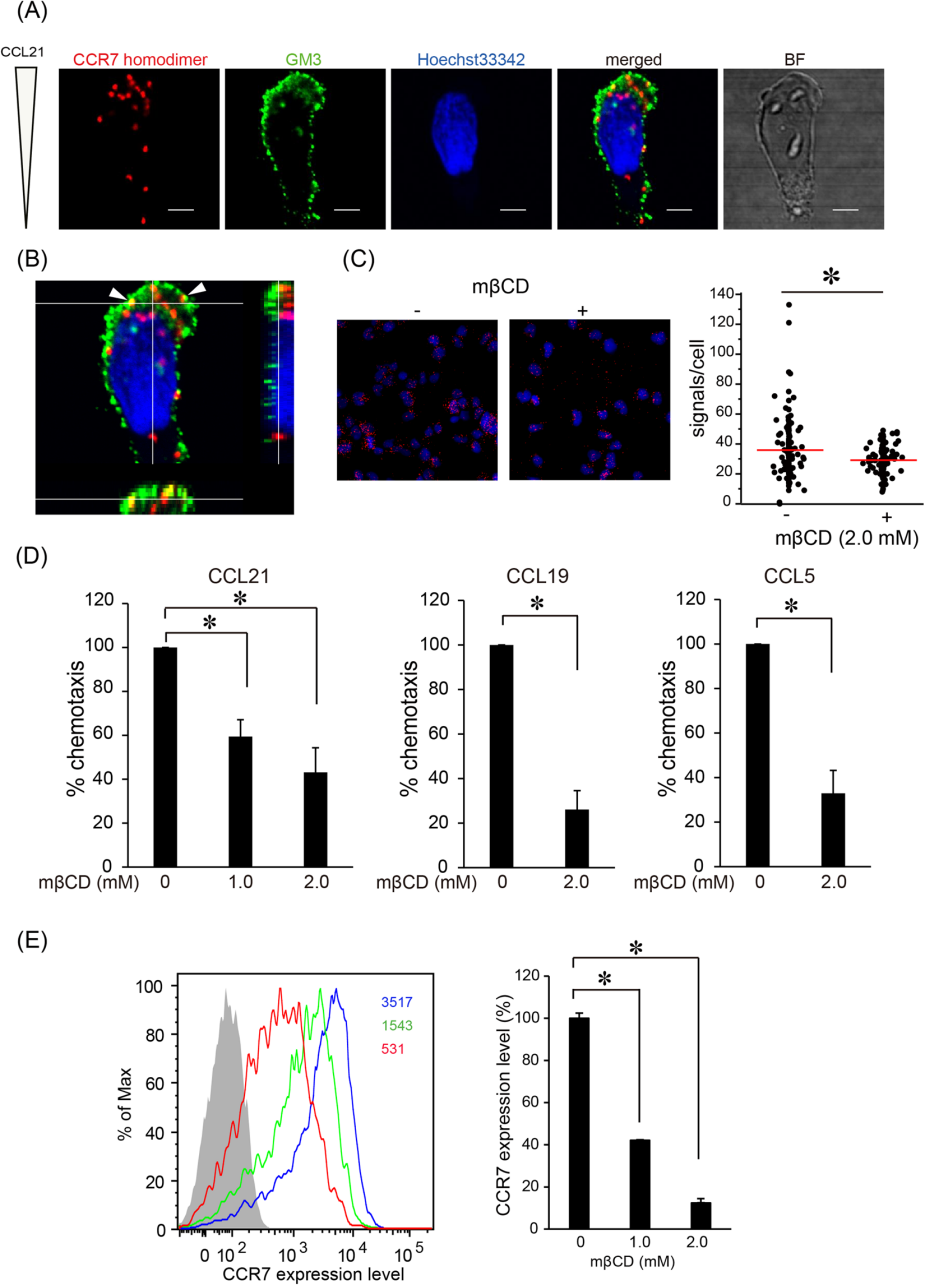
CCR7 homodimers are polarized toward GM3-enriched leading edge during CCR7-dependent cell migration. (**A**) The localization of CCR7 homodimers in T cell migration in response to CCL21 is shown (Red; PLA signal, Green; anti-GM3 antibody, Blue; Hoechst 33342). T cells were loaded into each well of the EZ-TAXIScan microchamber. After cell alignment was complete, human CCL21 (100 ng) was applied to the contra-wells. During migration, cells were fixed and stained with anti-human CCR7 antibody conjugated with the complementary oligonucleotide probes, anti-GM3 antibody, and Hoechst 33342. Scale bar: 10 μm. (**B**) Orthographic projection of the cell shown in (**A**). The center image illustrates a cross-sectional image (XY-plane), whereas the images to the right and below illustrate those in the YZ-plane and XZ-plane, respectively. White arrowheads show CCR7 homodimers detected in the GM3-enriched lipid rafts. (**C**) CCR7 homodimer formation after treatment with or without 2 mM MβCD was examined by *in situ* PLA (Red; PLA signal, Blue; Hoechst 33342). The number of PLA signals per cell was counted using the Duolink Image Tool software. The results shown are from one experiment, representative of three independent experiments, with the mean number of the signals plotted on the vertical axis. *p < 0.05 by Mann-Whitney’s U test. (**D**) Chemokine-induced cell migration with or without MβCD was examined using the Transwell assay. The indicated chemokine was added to the lower wells, and H9 cells were added to the upper wells in the presence of the indicated concentrations of MβCD. The results shown are from one experiment, representative of three independent experiments. Data represent mean ± SD of triplicate wells. *p < 0.05 by Student’s *t* test. (**E**) CCR7 expression levels were examined in the presence (red open histogram; 2 mM, green open histogram; 1 mM) or absence (blue open histogram) of MβCD by flow cytometric analysis using anti-human CCR7 antibody. The gray-filled histogram represents staining with isotype control antibody (left). The relative MFI of CCR7 expression is shown (right). The results shown are from one experiment, representative of three independent experiments. Data represent mean ± SD of triplicate samples. *p < 0.05 by Student’s *t* test.

We next investigated the localization of CCR7 homodimers in T cells migrating along the gradient of a CCR7 ligand chemokine. During CCL21-induced cell migration, CCR7 homodimers tended to polarize to the migrating front of cells (72.9 ± 12.6% of total), where ganglioside GM3, a component of cholesterol-based lipid microdomains, is predominantly accumulated (Fig. 2A and B, white arrowheads). When cholesterol was depleted with methyl-β-cyclodextrin (MβCD), CCR7 expression was reduced (Fig. 2E), and the levels of CCR7 homodimers also significantly decreased (Fig. 2C), indicating that cholesterol is required for stable CCR7 localization on the cell membrane. We also found that control CCR1 homodimer tended to polarize to the migrating front with GM3 during CCL5-mediated cell migration (Supplemental Fig. 7). MβCD treatment decreased CCR7-dependent cell migration in response to CCL21 or CCL19 (Fig. 2D) and also CCL5-induced CCR1-dependent cell migration without affecting cell viability (Supplemental Fig. 8), implying the possible contribution of membrane cholesterol to chemokine-induced cell migration in general.

### CCR7 homodimer formation is impaired by a peptide derived from the CCR7 transmembrane region 4

**Figure 3:**
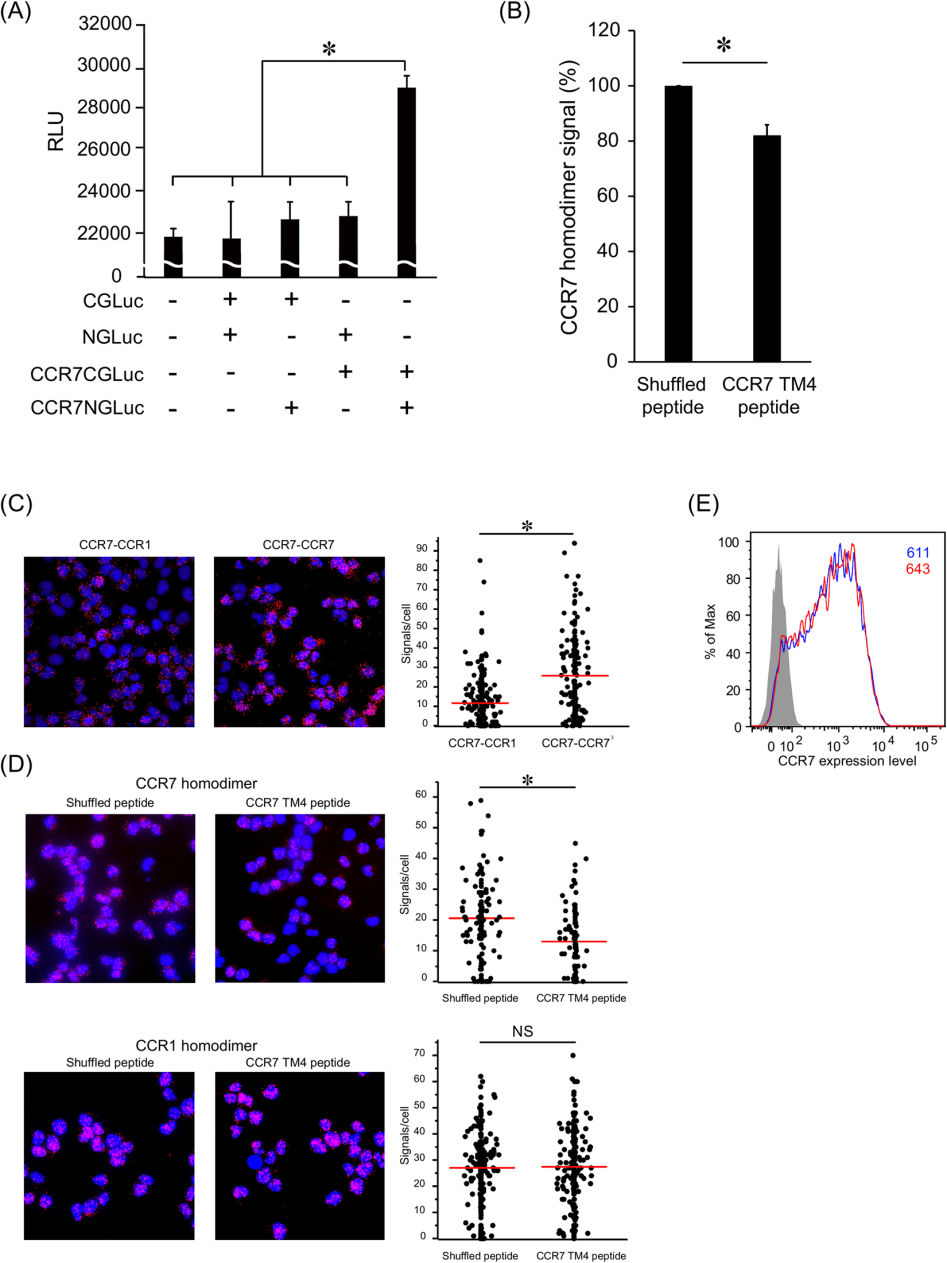
CCR7 homodimers are dissociated by the CCR7 TM4-derived peptide. (**A**) The levels of bioluminescence signals are presented for HEK293T cells transfected with the indicated combinations of CCR7-CGLuc, CCR7-NGLuc, CGLuc and NGLuc plasmids (1.5 μg each). A representative experiment from at least three independent experiments is shown. Data represent mean ± SD of triplicate samples. *p < 0.05 by one-way ANOVA. (**B**) The levels of bioluminescence signals are shown for cells transfected with combinations of CCR7-CGLuc and CCR7-NGLuc in the presence of the CCR7 TM4 peptide or the shuffled peptide (15 μg/ml). Relative luminescence was obtained by normalizing the values against untransfected cells. A representative experiment from at least three independent experiments is shown. Data represent mean ± SD of triplicate samples. *p < 0.05 by Student’s *t* test. (**C**) The levels of CCR7 homodimer in parental H9 cells were measured by *in situ* PLA. CCR7-CCR1 heterodimer formation detected by anti-CCR7 mAb-PLA^Plus^ and anti-CCR1 mAb -PLA^Minus^ (left), and CCR7 homodimer formation by anti-CCR7 mAb-PLA^Plus^ and -PLA^Minus^ (right). A representative experiment from at least three independent experiments is shown with the mean number of PLA signals plotted on the vertical axis. *p < 0.05 by Mann-Whitney’s U test. (**D**) CCR7 (top) or CCR1 (bottom) homodimer formation after treatment with the CCR7 TM4 peptide (right panel) or the shuffled peptide (left panel). The number of PLA signals per cell was counted using the Duolink Image Tool software. A representative experiment from three independent experiments is shown with the mean number of PLA signals plotted on the vertical axis. *p < 0.05 by Mann-Whitney’s U test; NS, not significant. (**E**) CCR7 expression levels were evaluated in the presence (red open histogram) or absence (blue open histogram) of the CCR7 TM4 peptide (15 μg/ml) by flow cytometry using anti-human CCR7 antibody or isotype control antibody (gray-filled histogram). MFI is indicated on the histograms. A representative experiment from three independent experiments is shown.

To further investigate the functional role of CCR7 homodimerization in CCR7 ligand-dependent cell migration and signaling, we designed a synthetic peptide that potentially inhibits CCR7 homodimer formation. Based on the previous reports that synthetic peptides corresponding to the fourth transmembrane region (TM4) of CXCR4 inhibited homodimer formation and CXCR4-dependent signaling^16^, we attempted to identify the corresponding CCR7 TM4 sequence by amino acid sequence alignment between the CCR7 and CXCR4 proteins using the T-Coffee algorithm^22^. We found that TM4 is the sequence from amino acids 166 to 195, and accordingly designed a CCR7 TM4-derived peptide, SCVGIWILATVLSIPELL, which shared a few amino acid residues with the CXCR4 TM4 peptide, VYVGVWIPALLLTIPDFI^16^. We then examined its effect on CCR7 homodimer formation in living cells with a split luciferase assay^23, 24^ using human embryonic kidney cell line 293 (HEK293T) cells expressing the fusion protein between the full-length human CCR7 and the N-terminal (CCR7-NGLuc) or the C-terminal (CCR7-CGLuc) half of GLuc. As shown in Fig. 3A, when treated with a substrate for GLuc, a strong bioluminescence signal was detected in the cells co-expressing CCR7-NGLuc and CCR7-CGLuc but not in mock-transfected cells, indicating that constitutive CCR7 homodimer formation was successfully detected using this system. As shown in Fig. 3B, the CCR7 TM4 peptide significantly decreased luminescence signals arising from the cells expressing CCR7 homodimer.

Next, we examined the effect of the CCR7 TM4 peptide on the endogenous CCR7 homodimer in H9 cells. CCR7 homodimer was readily detected in parental H9 cells by the combination of anti-CCR7 monoclonal antibody (mAb)-PLA^Plus^ and anti-CCR7 mAb-PLA^Minus^, whereas only weak signals were detected by the combination of anti-CCR7 mAb-PLA^Plus^/anti-CCR1 mAb-PLA^Minus^ or anti-CCR7 mAb-PLA^Minus^/anti-CCR1 mAb-PLA^Plus^ (Fig. 3C and Supplemental Fig. 9A), indicating that CCR7 homodimer is selectively detected by the PLA assay. When the cells were treated with the CCR7 TM4 peptide, the CCR7 homodimer level was significantly decreased (Fig. 3D, top panel and Supplemental Fig. 9B, left panel), whereas neither CCR1 nor CXCR4 homodimer level was significantly affected (Fig. 3D, bottom panel and Supplemental Fig. 9B, right panel). The cell surface CCR7 expression level was not affected by the presence of the CCR7 TM4 peptide (Fig. 3E), indicating that the CCR7 TM4 peptide selectively inhibited CCR7 homodimer formation without affecting the cell surface CCR7 expression level.

### CCR7-dependent cell migration is impaired by the CCR7 TM4-derived peptide

**Figure 4:**
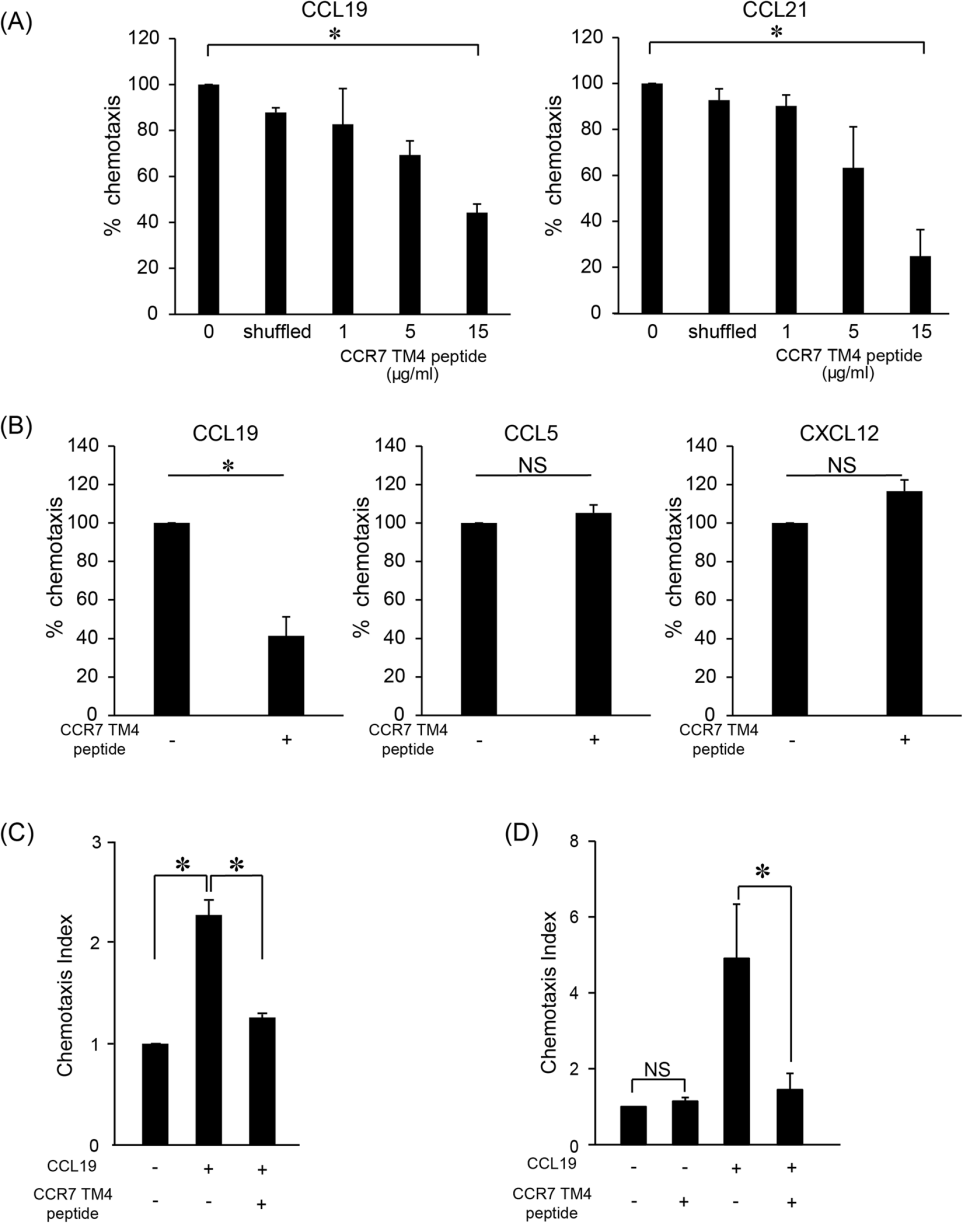
CCR7-dependent cell migration is inhibited by the CCR7 TM4 peptide. (**A**) CCR7-dependent T cell chemotaxis in the presence of the CCR7 TM4 peptide was examined using the Transwell assay. CCL19 (100 ng/ml, left panel) or CCL21 (100 ng/ml, right panel) was added to the lower wells, and cells premixed with the indicated concentrations of the CCR7 TM4 peptide or 15 μg/ml of the shuffled peptide were added to the upper wells. The result shown is a representative one from three independent experiments. Data represent mean ± SD of triplicate wells. (**B**) T cell chemotaxis in response to 100 ng/ml CCL19, CXCL12, or CCL5 was examined using the Transwell assay. Each chemokine was added to the lower well, and cells mixed with or without the CCR7 TM4 peptide (15 μg/ml) were added to the upper well. The percentages of input cells that had migrated to the lower well are shown. The result shown is a representative one from three independent experiments. Data represent mean ± SD of triplicate wells. (**C**) The level of MDA231 cell migration is shown in response to 100 ng/ml CCL19 with or without the CCR7 TM4 peptide (15 μg/ml). The number of cells that migrated to the bottom wells of transwell chambers were counted by Hoechst 33342 staining. Data represent mean ± SEM of triplicate experiments. (**D**) Primary human T cell chemotaxis in response to CCL19 in the presence or absence of the CCR7 TM4 peptide (15 μg/ml). The result shown is a representative one from three independent experiments. Data represent mean ± SD of triplicate wells. *p < 0.05 by Student’s *t* test; NS, not significant.

We next examined the effect of the CCR7 TM4 peptide on CCR7 ligand-dependent cell migration. As shown in Fig. 4A, the CCR7 TM4 peptide, but not a shuffled peptide, inhibited CCR7 ligand-induced T cell migration in a dose-dependent manner. In the same setting, the CCR7 TM4 peptide did not affect CXCL12- or CCL5-induced T cell migration (Fig. 4B), indicating that the TM4 peptide acted specifically on CCR7. As shown in Supplemental Fig. 10A, the inhibitory effect of the CCR7 TM4 peptide was clearly observed in cell migration under 10–100 ng/ml CCL19 concentrations, whereas only marginally under 500 ng/ml. We next examined the effect of the CCR7 TM4 peptide on CCR7-dependent response in MDA-MB-231 (MDA231) human breast cancer cells and in primary human T cells. We found that the TM4 peptide significantly impaired CCL19-induced cell migration in both MDA231 (Fig. 4C) and primary T cells (Fig. 4D). The TM4 peptide also impaired CCL21-dependent cell migration in these cells (Supplemental Fig. 10B and C). These results further strengthen the hypothesis that CCR7 homodimerization plays a critical role in CCR7-dependent cell migration in multiple cell types.

### Both CCL19-Ig binding and CCL19-induced CCR7 internalization are decreased by the CCR7 TM4 peptide

**Figure 5:**
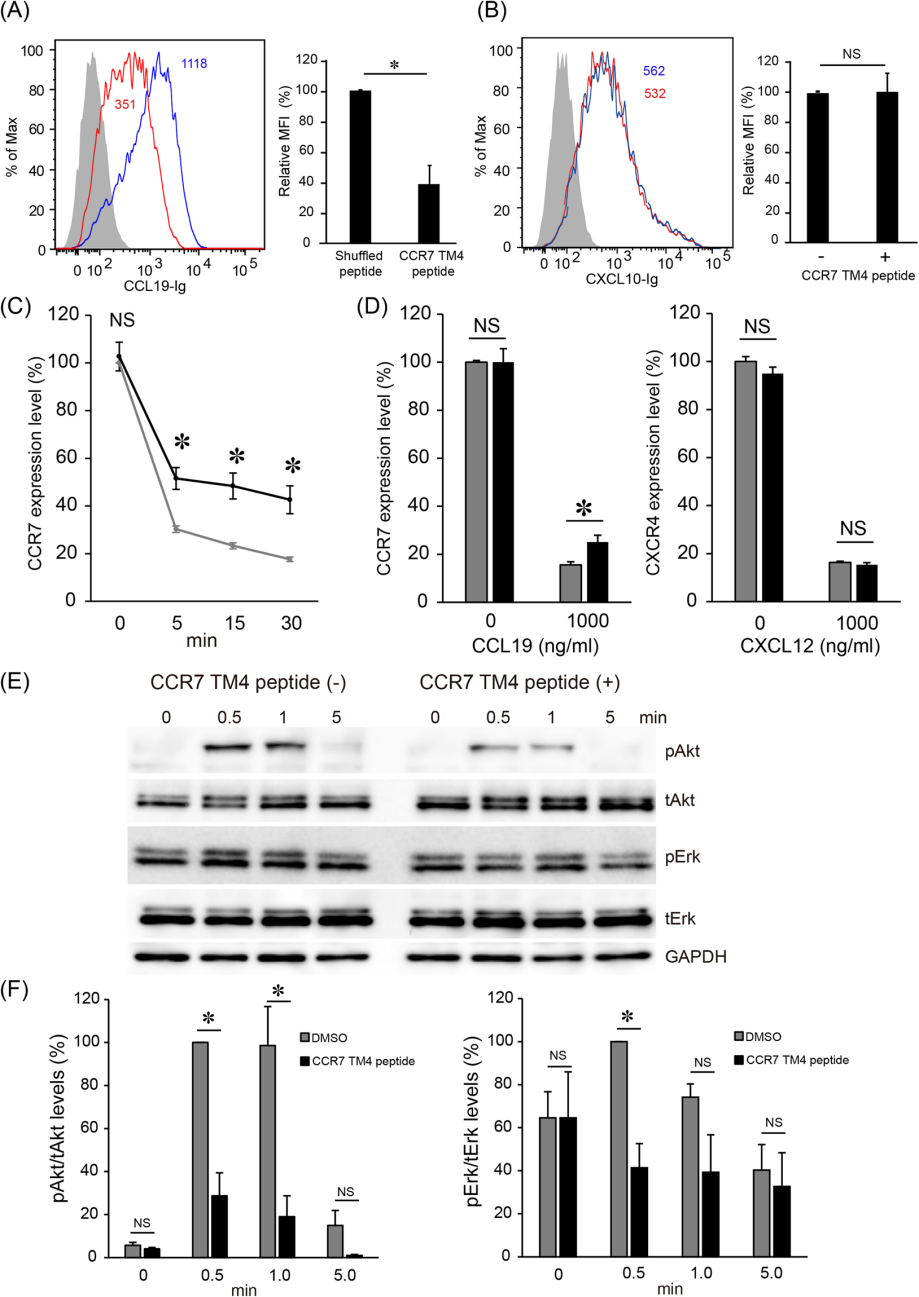
CCL19 binding, CCL19-induced CCR7 internalization and CCR7-dependent signaling are inhibited by the CCR7 TM4 peptide. (**A**) Flow cytometric analysis of CCL19-Ig (100 ng/ml) binding in H9 cells with the CCR7 TM4 peptide (red open histogram) or the shuffled peptide (blue open histogram). The gray-filled histogram shows the staining with control immunoglobulin. MFI is indicated on the histograms (left). The relative MFI of CCL19-Ig binding with the CCR7 TM4 peptide or the shuffled peptide is shown (right). Data are mean ± SD of three independent experiments. (**B**) Flow cytometric analysis of CXCL10-Ig binding with (red open histogram) or without (blue open histogram) the CCR7 TM4 peptide. The gray-filled histogram shows the staining with control immunoglobulin. MFI is indicated on the histogram (left). The relative MFI of CXCL10-Ig binding is shown (right). Data are mean ± SD of three independent experiments. (**C**) CCR7 expression levels after addition of 1 μg/ml CCL19 were evaluated in the presence (black line) or absence (gray line) of the CCR7 TM4 peptide by flow cytometry using anti-CCR7 antibody. Data are mean ± SD of three independent experiments. (**D**) CCR7 (left) or CXCR4 (right) expression levels after addition of the indicated chemokine for 30 minutes were evaluated in the presence (black bars) or absence (gray bars) of the CCR7 TM4 peptide by flow cytometry using anti-human CCR7 antibody or anti-human CXCR4 antibody. (**E**) CCR7 ligand-induced phosphorylation of Akt and p44/42 MAPK (Erk1/2) at the indicated time points after 1 µg/ml CCL19 treatment with or without 15 μg/ml CCR7 TM4 peptide. The levels of phosphorylated and total Akt and Erk1/2 were analyzed by Western blotting using antibodies against phosphorylated Akt at Thr308 (pAkt), phosphorylated Erk1/2 (pErk), total Akt (tAkt), total Erk1/2 (tErk) or GAPDH. A representative experiment from three independent experiments is shown. (**F**) The ratio of pAkt/tAkt (left) and pErk/tErk (right) were analyzed by densitometric analysis. Data are expressed as percentage of pAkt or pErk levels of control treatment at 0.5 min. Data are mean ± SEM of three independent experiments. *p < 0.05 by Student’s *t* test; NS, not significant.

We next assessed the contribution of CCR7 homodimerization to ligand binding. As shown in Fig. 5A, the CCR7 TM4 peptide decreased CCL19-Ig binding by approximately 60%. In contrast, the TM4 peptide did not affect CXCL10-Ig binding, indicating that the peptide decreased CCL19 binding specifically (Fig. 5B). These results support the idea that CCR7 homodimerization promotes CCR7 signaling by increasing ligand binding.

To further assess the contribution of CCR7 homodimerization to CCR7-dependent signaling, we next examined the effect of the CCR7 TM4 peptide on CCL19-induced CCR7 internalization. When cells were stimulated with CCL19, the cell surface CCR7 was rapidly internalized to the cytoplasm, showing a 70% decrease in cell surface CCR7 expression within 5 minutes (Fig. 5C). In the presence of the CCR7 TM4 peptide, the reduction of the cell surface CCR7 expression was much less pronounced, indicating that the CCR7 TM4 peptide decreased CCL19-induced CCR7 internalization effectively (Fig. 5C and D, left panel). In contrast, the CCR7 TM4 peptide did not affect CXCL12-induced CXCR4 internalization (Fig. 5D, right panel), indicating that the peptide selectively acted on CCR7. These results further confirm that CCR7 homodimerization contributes to the CCR7-dependent signaling.

### CCR7 ligand-induced Akt and Erk signaling and actin rearrangement are inhibited by the CCR7 TM4-derived peptide

We next examined the effect of the CCR7 TM4 peptide on ligand-induced Akt and Erk phosphorylation. As shown in Fig. 5E and F, Akt was transiently phosphorylated with a peak at 0.5–1 min after CCL19 treatment, and returned to the basal level by 5 min. In the presence of the CCR7 TM4 peptide, the peak Akt phosphorylation levels were strongly reduced. Similarly, Erk was transiently phosphorylated with a peak at 0.5–1 min after CCL19 treatment, whereas, in the presence of the CCR7 TM4 peptide, Erk phosphorylation remained at basal levels throughout the course of the experiment. CCL21-induced phosphorylation of Akt and Erk were also impaired by the CCR7 TM4 peptide, albeit to a lesser extent (Supplemental Fig. 11). These results indicate that CCR7 homodimerization facilitates CCR7-dependent intracellular signaling. Since chemokine-mediated signaling is known to induce actin rearrangements during cell polarization and migration towards the chemokine gradient^25^, we next examined the effect of the CCR7 TM4 peptide on CCR7 ligand-induced actin polymerization. We found that CCL19 treatment rapidly increased filamentous actin (F-actin) levels within 0.5 min, which was significantly impaired by the CCR7 TM4 peptide (Supplemental Fig. 12).

## Discussion

In this report, we demonstrated the critical contribution of CCR7 homodimerization to CCR7-dependent cell migration and signaling. Previous studies demonstrated that chemokine receptors including CCR2^26^, CXCR4^27^, or CCR5^28^, and more recently CCR7^6, 18^, form constitutive or ligand-induced homo- and/or heterodimers; however, the exact contribution of the receptor dimers to signal transduction has not been elucidated. In this study, we used a model system that allows artificial induction of CCR7 homo- or heterodimerization and showed that CCR7 homodimerization rather than CXCR4/CCR7 heterodimerization facilitates CCR7 signaling. Destabilizing CCR7 homodimerization with the CCR7 TM4 peptide, an 18-residue peptide corresponding to the fourth TM domain, inhibited CCR7-dependent signaling, indicating that CCR7 homodimerization occurring via a TM4 interface plays a role in optimal functioning of CCR7. Similar observations have also been reported for CXCR4^16^ and CCR5^15^, and in another class of GPCR, glucagon-like peptide-1 receptor^29^, where the TM4 interface is shown to be essential for the maintenance of a high-affinity G protein-complexed state. Given that the TM4 domains of CXCR4 and CCR7 share 43% amino acid sequence identity and 70% similarity and that those of CCR5 and CCR7 share 23% amino acid sequence identity and 67% similarity, the TM4 domain of chemokine receptors may share a general dimerization motif. In addition to the TM4 interface, a recent report has suggested the importance of another hydrophobic dimer interface comprising the end of TM7 and helix 8 of CCR7^18^, suggesting that multiple TM interfaces are involved in dimerization and assembly of the CCR7 molecules. Several possibilities can be considered with regard to the mechanistic basis for increased ligand binding by CCR7 homodimers. First, the dimeric molecules may work cooperatively to provide an interface required for enhanced CCL19 binding. Second, receptor dimerization may induce an allosteric change in the ligand binding site of the receptor such that CCL19 binds better. Although the TM4 peptide inhibited CCR7 homodimer formation only modestly in the *in situ* PLA assay and split luciferase assay, disruption of the TM4 interface may have still allowed homodimer-like receptor aggregation that emitted a positive PLA signal but effectively abrogated the dimer interface required for optimal function of the receptor^28^, thereby attenuating ligand binding and assembly of signaling complexes responding to CCR7 ligands. It may also be that the TM4 peptide induces a conformational change in CCR7 homodimer and/or in the ligand binding domain of CCR7, thereby impairing CCR7-dependent signaling.

CCR7 homodimerization also appears to enhance receptor-mediated signaling by inducing accumulation of CCR7 in the sphingolipid- and cholesterol-rich lipid raft microdomains that selectively sequester the signaling machinery necessary for inducing cell migration^30, 31^. Previous studies showed that CXCR4 and CCR5 chemokine receptors are preferentially localized and activated in highly organized lipid raft domains that are required for their efficient signal transduction^32, 33^. We showed here that CCR7 homodimers were polarized in the GM3-rich membrane raft domains at the front of cells migrating along the CCR7 ligand gradient. Disruption of CCR7 homodimers by cholesterol depletion resulted in a marked decrease in CCR7 ligand-induced cell migration, in agreement with the idea that CCR7 homodimerization at lipid rafts provides an essential scaffold for efficient signal delivery through CCR7.

We showed that T cell chemotaxis in response to low CCR7 ligand concentrations was facilitated by induction of CCR7 homodimerization, suggesting that T cells may migrate efficiently through CCR7 homodimerization, when CCR7-ligand availability is low *in vivo*. Whereas CCR7 ligand concentrations of 100 ng/ml (i.e. about 10 nM) or greater were required for efficient T cell migration *in vitro* in this study, the reported CCR7 ligand concentrations *in vivo* are indeed much lower. The plasma levels of CCL19 and CCL21 are about 30–90 pg/ml and 500 pg/ml, respectively, in healthy individuals^34^. Even under pathological conditions, CCL21 levels in the plasma of carotid atherosclerosis patients^35^ and synovial fluids from rheumatoid arthritis patients^36^ are approximately 1,000 pg/ml. Given however that deficiency in CCR7 or its ligands results in severe immunological outcomes *in vivo* ^1, 3^, it would be reasonable to assume a mechanism whereby CCR7 would function properly even at low ligand concentrations *in vivo*. We have previously shown that CXCR4 binding molecules, including CXCL12^6, 37^ and HIV-1 gp120^6^, can enhance CCR7 sensitivity and CCR7 homodimerization. Other groups have shown that prostaglandin E2 can enhance CCR7-dependent signal transduction through receptor dimerization in dendritic cells^18, 38^. These results lead us to propose that sensitivity of CCR7 to its ligand chemokines can be facilitated by a number of tissue-derived endogenous factors that induce CCR7 dimerization *in vivo*.

In this study, we provide new insights into the regulation of CCR7-dependent signaling through CCR7 homodimer formation. Given that the level of CCR7-dependent signaling was accompanied by CCR7 homodimerization in multiple cell types including primary human T cells and breast cancer cells, CCR7-dependent cell migration may be controlled by CCR7 homodimerization in various biological contexts, such as lymphocyte trafficking^1, 3^ and CCR7-dependent metastatic progression in tumors^39–41^. Further study of raft-associated signaling components interacting with the CCR7 homodimer and of the endogenous factors controlling the molecular equilibrium of CCR7 between monomeric and dimeric states will be required to understand the precise molecular mechanisms and functions of CCR7 homodimer formation.

## Methods

### Cells

The human CD4^+^ T cell line, H9 (HTB-176), and HEK293T were obtained from the American Type Culture Collection. The human breast cancer cell line MDA231 was obtained from the Osaka Medical Center for Cancer and Cardiovascular Diseases. Peripheral blood mononuclear cells (PBMCs) were obtained from healthy donors with written informed consent under the protocol approved by the Ethics Review Committee of Kindai University (No. 16-001). The cells were cultured in Roswell Park Memorial Institute (RPMI) 1640 medium or Dulbecco’s Modified Eagle Medium (DMEM; Wako Pure Chemica, Osaka, Japan) supplemented with 10% (v/v) Fetal Bovine Serum (FBS) (PAA Laboratories, Pasching, Austria), 2 mM L-glutamine, 1 mM sodium pyruvate, 100 U/ml penicillin, 100 μg/ml streptomycin, 0.1 mM nonessential amino acids (all from Wako Pure Chemical), 50 μM 2-mercaptoethanol (Thermo Fisher Scientific, MA, USA), and 10 mM HEPES (GE Healthcare Life Sciences, Little Chalfont, UK). For serum starvation, H9 cells or MDA231 cells were washed three times and cultured in RPMI1640 medium or DMEM containing 0.1% Bovine serum albumin (BSA; Sigma, MO, USA) for 30 min before use.

The CXCR4/CCR7-knockout H9 cell line was established by the CRISPR/Cas9 genome editing system (Invitrogen, CA, USA) according to the manufacturer’s instructions. The guide sequences for the CCR7 knockout were 5′-AATGAAAAGCGTGCTGGTGGGTTTT-3′ and 5′-CCACCAGCACGCTTTTCATTCGGTG-3′, whereas those for the CXCR4 knockout were 5′-CACTTCAGATAACTACACCGGTTTT-3′ and 5′-CGGTGTAGTTATCTGAAGTGCGGTG-3′. The vectors harboring these guide sequences were transfected into H9 cells using the Amaxa Cell Line Nucleofector Kit V (Lonza, Basal, Switzerland). After cell expansion, orange fluorescent protein-expressing cells were enriched using a FACSAriaII cell sorter (BD Biosciences, CA, USA).

The stably transfected H9-derived cell lines were established as follows. To obtain recombinant retroviral particles for H9 cell infection, the HEK293T cells were seeded at a density of 5 × 10^6^ cells per 10-cm diameter plastic tissue culture dish, and were transiently transfected using PEI-MAX (Polysciences, PA, USA) with the pCL-Ampho retrovirus packaging vector (15 μg) and the pCX4bsr-CCR7DmrA, pCX4bsr-CXCR4DmrA or pCX4EGFP-CCR7DmrC (15 μg) plasmid for 12 h. For production of retroviral particles, the transfected HEK293T cells were cultured in fresh media for a further 24 h. The virus-containing medium was collected, centrifuged at 1,700 rpm for 90 min at 25 °C, and the supernatant added to H9 cells pretreated with 8 μg polybrene (Santa Cruz Biotechnology, CA, USA). The CCR7 and/or CXCR4-expressing population of H9 cells was selected for resistance to 10 μg/ml blasticidin for two weeks and/or enriched using a FACSAriaII cell sorter.

### Plasmids

The *Gaussia* luciferase (GLuc) expressing vector (pTKGLuc) was purchased from New England Biolabs (UK) Ltd. (Hertfordshire, UK). The DmrA (FK506 binding protein) or DmrC (FKBP-rapamycin binding domain) expressing vectors (pHet-Mem1 or pHet-1, respectively) were purchased from Clontech (CA, USA). To establish N-terminal (NGLuc) and C-terminal (CGLuc) fragments of GLuc for fusion with CCR7, the NGLuc fragment was amplified with primers including a single NotI site: 5′-GGTGGAGGCGGTTCAGGTGGAGGCGGTTCAGCCAAGCCCACCGAGAACAACG-3′ and 5′-AAAAAGCGGCCGCTTAGCCTATGCCGCCCTGTGCGG-3′. The CGLuc fragment was amplified with PCR primers incorporating a single NotI site: 5′-GGTGGAGGCGGTTCAGGTGGAGGCGGTTCAGAGGCGATCGTCGACATTCC -3′ and 5′-AAAAAGCGGCCGCTTAGTCACCACCGGCCCCCT-3′. The human CCR7 was amplified with PCR primers including the EcoRI site: 5′-TTTTTGAATTCAGAGAGCGTCATGGACCTGGGGAAACCAAT-3′ and 5′-TGAACCGCCTCCACCTGAACCGCCTCCACCTGGGGAGAAGGTGGTGGTGGTC-3′. The CCR7 and NGLuc or CGLuc fragments were then assembled using secondary PCR, and were subcloned into corresponding EcoRI and NotI sites of the pEGFP-N1 vector (Clontech).

To construct the CCR7-DmrA, CCR7-DmrC, and CXCR4-DmrA expression vectors, human CCR7 or CXCR4 cDNAs were amplified using PCR and subcloned into corresponding EcoRI and XbaI sites of the pHet-1 or pHet-Mem1plasmids. The full-length human CCR7 cDNA was amplified with a pair of PCR primers 5′-TTTTTGAATTCAGAGAGCGTCATGGACCTGGGGAAACCAAT-3′ and 5′-TTTTTTCTAGATGGGGAGAAGGTGGTGGTGG-3′ including EcoRI and XbaI sites, respectively. The full-length human CXCR4 cDNA was amplified with a pair of PCR primers 5′- TTTTTGAATTCATGGAGGGGATCAGTATATA-3′ and 5′-TTTTTTCTAGAGCTGGAGTGAAAACTTGAAG-3′ including EcoRI and XbaI sites, respectively. The CCR7-DmrA and CCR7-DmrC were amplified with a pair of PCR primers 5′-TTTTTGAATTCAGAGAGCGTCATGGACCTGGGGAAACCAAT-3′ and 5′-TTTTTGCGGCCGCTTATGCGTAGTCTGGTACGTCGTACG-3′ including EcoRI and NotI sites, respectively. The CXCR4-DmrA was amplified with a pair of PCR primers 5′-TTTTTGAATTCATGGAGGGGATCAGTATATA-3′ and 5′-TTTTTGCGGCCGCTTATGCGTAGTCTGGTACGTCGTACG-3′ including EcoRI and NotI sites, respectively. The PCR products were subcloned into corresponding EcoRI and NotI sites of the retroviral vector pCX4bsr^42^ or pCX4EGFP plasmids.

### Synthetic peptides

The CCR7 TM4 peptide (SCVGIWILATVLSIPELL) and a shuffled peptide (AILLTCILPSVEILSVWG), in which the amino acid sequence of the CCR7 TM4 peptide is rearranged randomly, were purchased from Thermo Fisher Scientific or synthesized using the solid-phase method^43^. The peptide was dissolved in a small amount of DMSO, and then diluted with water to the designed concentration.

### Split Gaussia luciferase assay

A substrate for GLuc, coelenterazine h, was purchased from Wako Pure Chemicals. HEK293T cells (5 × 10^5^ cells per well) were cultured in 6-well dishes for 24 h, and transiently transfected with plasmids encoding CCR7-NGLuc and CCR7-CGLuc using PEI-MAX. After 24 h, the cells were cultured in fresh media for a further 24 h and subjected to the luminescence measurement. Five minutes after adding coelenterazine h (20 μM) to phenol red-free culture medium, luminescence signals were integrated over 2 sec using a LB9507 luminometer (Berthold Technologies, Bad Wildbad, Germany) or over 10 sec using an IVIS system (Perkin Elmer, MA, USA).

### CCL19-Ig binding assay

Cells were pretreated with or without synthetic peptides (15 μg/ml) or A/C Heterodimerizer (Clontech), and then stained with 1 μg/ml CCL19-Ig (eBioscience, CA, USA) for 30 min, followed by PE-labeled anti-human IgG for 20 min on ice. CCL19-Ig binding levels were measured with a FACSVerse or FACSFortessa flow cytometer (BD Biosciences) and the data analyzed with FlowJo software (Tree Star Inc., OR, USA).

### Chemokine receptor internalization assay

The serum-starved H9 cells (100 μl of a suspension at 1 × 10^7^ cells/ml) were pretreated with or without synthetic peptides (15 μg/ml) for 30 min at 37 °C, and then treated with CCL19 or CXCL12 (R&D Systems, MN, USA) at 100–1,000 ng/ml for 0–30 min at 37 °C. The reaction was stopped by adding a 10-fold excess volume of ice-cold PBS containing 0.1% BSA to the cells. The cell surface CCR7 or CXCR4 levels were detected with biotin-conjugated anti-CCR7 mAb 3D12 (BD Biosciences), followed by DyLight649-conjugated streptavidin (Biolegend, CA, USA) or APC-conjugated anti-CXCR4 mAb 12G5 (Biolegend). The data acquisition and analysis were carried out using a FACSVerse flow cytometer or FACS AriaII cell sorter. The expression levels of cell surface CCR7 or CXCR4 receptors as measured by fluorescence intensity were standardized against isotype control values, and expressed as a percentage in relation to the peptide-free condition.

### Transwell cell migration assay

The serum-starved H9 cells were treated with or without NSC87877, PHPS1, PTX (all from Calbiochem, CA, USA), synthetic peptides or A/C Heterodimerizer for 30 min at 37 °C, and were added to the upper chamber of a 24-well plate containing Transwell inserts with a pore size of 8.0 μm (Corning, NY, USA). Human CCL19, CCL21, CXCL12 or CCL5 (R&D Systems) were applied to the lower chamber in RPMI1640 medium containing 0.1% BSA. After 2 h incubation at 37 °C in a CO_2_ incubator, the number of cells that had migrated into the lower chamber was counted with a FACSverse, FACSFortessa flow cytometer or FACS AriaII cell sorter. For primary human T cells, serum-starved PBMCs were added to the upper chamber of a 24-well Transwell plate containing inserts with a 5.0 μm pore size. After incubation for 2 h at 37 °C in a CO_2_ incubator, the cells that had migrated into the lower chamber in response to CCL19 or CCL21 were stained with PE-conjugated anti-human CD4 mAb and FITC-conjugated anti-human CD8 mAb (BD Bioscience) for 20 min on ice. CD4^+^ and CD8^+^ T cells were counted using a FACSFortessa flow cytometer. For MDA231 cells, the lower surface of Transwell inserts with a 5.0 μm pore size was pre-coated with fibronectin (Sigma-Aldrich, MO, USA) for 1 h at room temperature, and washed two times in PBS and dried for 1 h. Serum-starved MDA231 cells in DMEM containing 0.1% BSA were added to the upper chamber, and CCL19 or CCL21 in the same medium was added to the lower chamber. After incubation for 4 h at 37 °C in 5% CO_2_, the cells that migrated through the filters were fixed using 4% paraformaldehyde (PFA; Wako Pure Chemical) and stained with Hoechst 33342 (Sigma-Aldrich). Fluorescent images were captured using a fluorescence microscope at 4x magnification (Olympus IX71), and the number of migrated cells was analyzed with Duolink Image Tool software (Sigma), a software for objective quantification in single cell image, where the nuclei and cytoplasm were automatically detected, and PLA signals generated were counted.

### PLA

CCR7 homodimer formation was examined using PLA as described previously^6^. Briefly, serum-starved H9 cells (200 μl of a suspension with 1 × 10^6^ cells/ml) in RPMI1640 medium/0.1% BSA were treated with or without synthetic peptides (15 μg/ml) or 500 nM A/C Heterodimerizer for 30 min at 37 °C in an 8-well chamber slide (BD Biosciences or Merck, Darmstadt, German). The cells were fixed with 4% PFA for 10 min, and stained with 7.5 μg/ml anti-human CCR7 mAb (MAB197; R&D Systems) or anti-human CCR1 mAb (clone 53504; R&D Systems) conjugated with either the PLA^Minus^ or PLA^Plus^ probes generated with Duolink II Probemaker (Sigma). The images were acquired using a confocal laser microscope (FV1000, FV1000-D or LSM710) or a fluorescence microscope (BX50), and dimerized CCR7 observed as orange dots was quantified using the Duolink Image Tool software. The percentage of CCR7 dimers present in the GM3-positive vs. -negative areas, was calculated in 20 different cells by the analysis using ImageJ software (NIH, MD, USA).

### Western blotting

H9 cells cultured overnight in RPMI1640 medium/0.1% BSA were treated with CCL19 or CCL21 at 37 °C, and then washed with media containing ice-cold PhosStop (Roche, Basal, Switzerland). The cells were lysed by adding 50 mM Tris-HCl, pH 7.4, 150 mM NaCl, 1 mM EDTA, 1% Triton-X, PhosStop and Protease Inhibitor Tablets (Sigma). The samples were separated by SDS-PAGE, and transferred onto PVDF membranes for immunoblotting with an anti-phospho-Akt, phospho-Erk1/2, total-Akt, total-Erk1/2 antibody (Cell Signaling Inc., MA, USA) or anti-GAPDH antibody (Biolegend). Bound antibodies were detected using the ECL system (GE Healthcare Life Sciences).

### Actin rearrangement

The serum-starved H9 cells (100 μl of a suspension at 1 × 10^7^ cells/ml) were pretreated with or without synthetic peptides (15 μg/ml) for 30 min at 37 °C, and then treated with CCL19 at 1 µg/ml for 0–5 min at 37 °C. The reaction was stopped by adding a 5-fold excess volume of ice-cold 4% PFA for 30 min. Cells were permeabilized with ice-cold 0.1% Triton-X (Sigma-Aldrich) for 1 min, and then stained with Alexa Flour 647-conjugated phalloidin (Invitrogen) for 30 min. The data acquisition and analysis were carried out using FACS AriaII cell sorter. Actin polymerization levels as measured by fluorescence intensity were expressed as a percentage in relation to the peptide-free condition.

### Ethics statement

The experimental protocols were approved by the Ethics Review Committee of Kindai University. All experiments were conducted in accordance with approved guidelines of Kindai University.

## Electronic supplementary material

Supplemental figure
