# Supplementary material for: Regulation of CCR7-dependent cell migration through CCR7 homodimer formation
Source: Sci Rep. 2017 Aug 17;7:8536. doi: 10.1038/s41598-017-09113-4 (PMC5561199; doi:10.1038/s41598-017-09113-4)
Supplement: Supplementary file 1 — Supplemental figure [file 41598_2017_9113_MOESM1_ESM.pdf]

Regulation of CCR7-dependent cell migration through CCR7 homodimer formation

Daichi Kobayashi[1,2,6], Masataka Endo[2], Hirotaka Ochi[2], Hironobu Hojo[3], Masayuki Miyasaka[4,5,6], Haruko Hayasaka[2*]

[1]Laboratory of Immune Regulation, Department of Microbiology and Immunology, Osaka University Graduate School of Medicine, 2-2 Yamada-oka, Suita, Osaka 565-0871, Japan

[2]Laboratory of Immune Molecular Function, Faculty of Science & Engineering, Kindai University, 3-4-1 Kowakae, Higashiosaka, Osaka 577-8502, Japan

[3]Institute for Protein Research, Osaka University, 3-2 Yamada-oka, Suita, Osaka 565-0871, Japan

[4]Interdisciplinary Program for Biomedical Sciences, Institute for Academic Initiatives, Osaka University, 2-2 Yamada-oka, Suita, Osaka 565-0871, Japan

[5]MediCity Research Laboratory, University of Turku, FIN-20520 Turku, Finland

[6]WPI Immunology Frontier Research Center, Osaka University, 2-2 Yamada-oka, Suita, Osaka 565-0871, Japan

[*]Corresponding author: hhayasaka@life.kindai.ac.jp

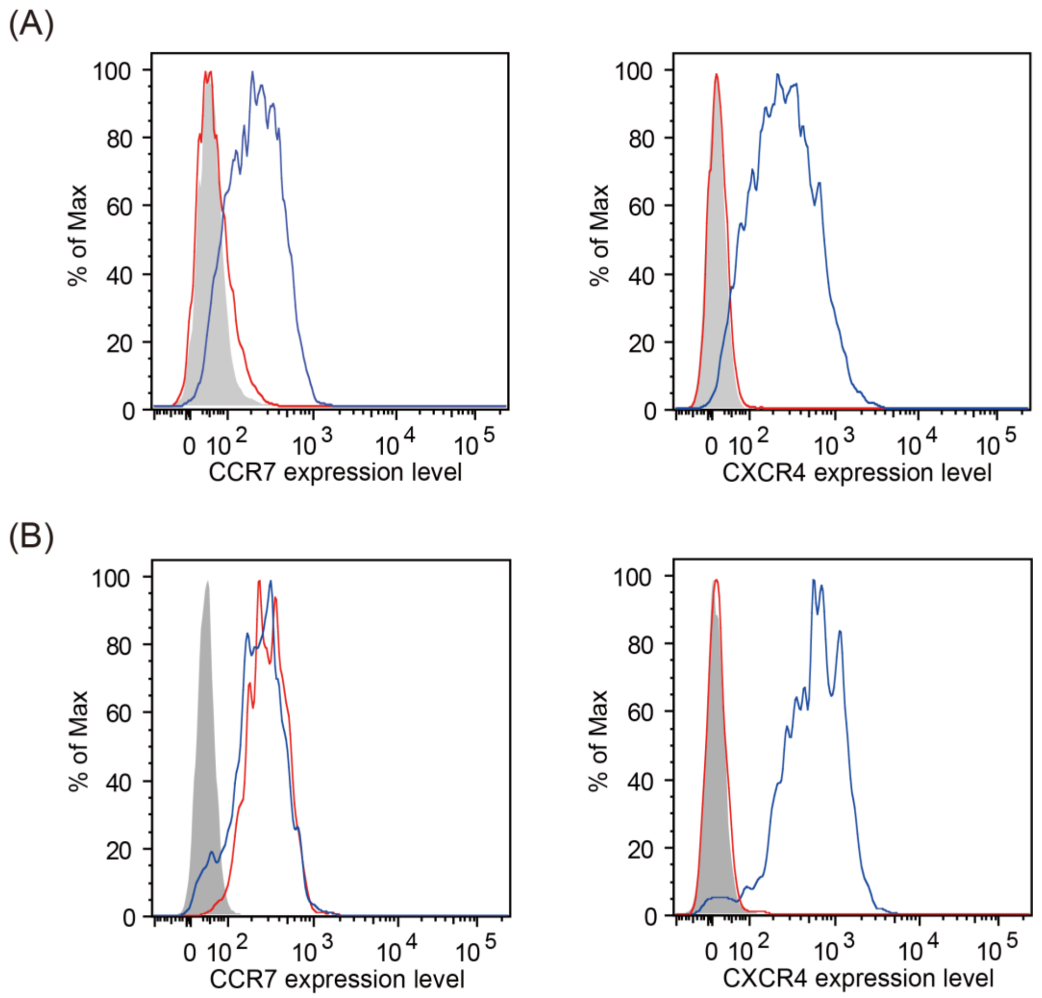

Supplemental figure 1. Flow cytometric analysis of CCR7 and CXCR4 expression in the H9 cell lines. (A) Parental H9 cells (blue) and CXCR4/CCR7 knockout H9 cells established by the CRISPR/Cas9 system (red) were stained with anti-CCR7 (left panel), anti-CXCR4 (right panel), or isotype control (shaded gray histogram) antibody.

(B) CCR7-DmrA/CCR7-DmrC (red) and CXCR4-DmrA/CCR7-DmrC cells (blue) were stained with anti-CCR7 (left panel), anti-CXCR4 (right panel), or isotype control (shaded gray histogram) antibody.

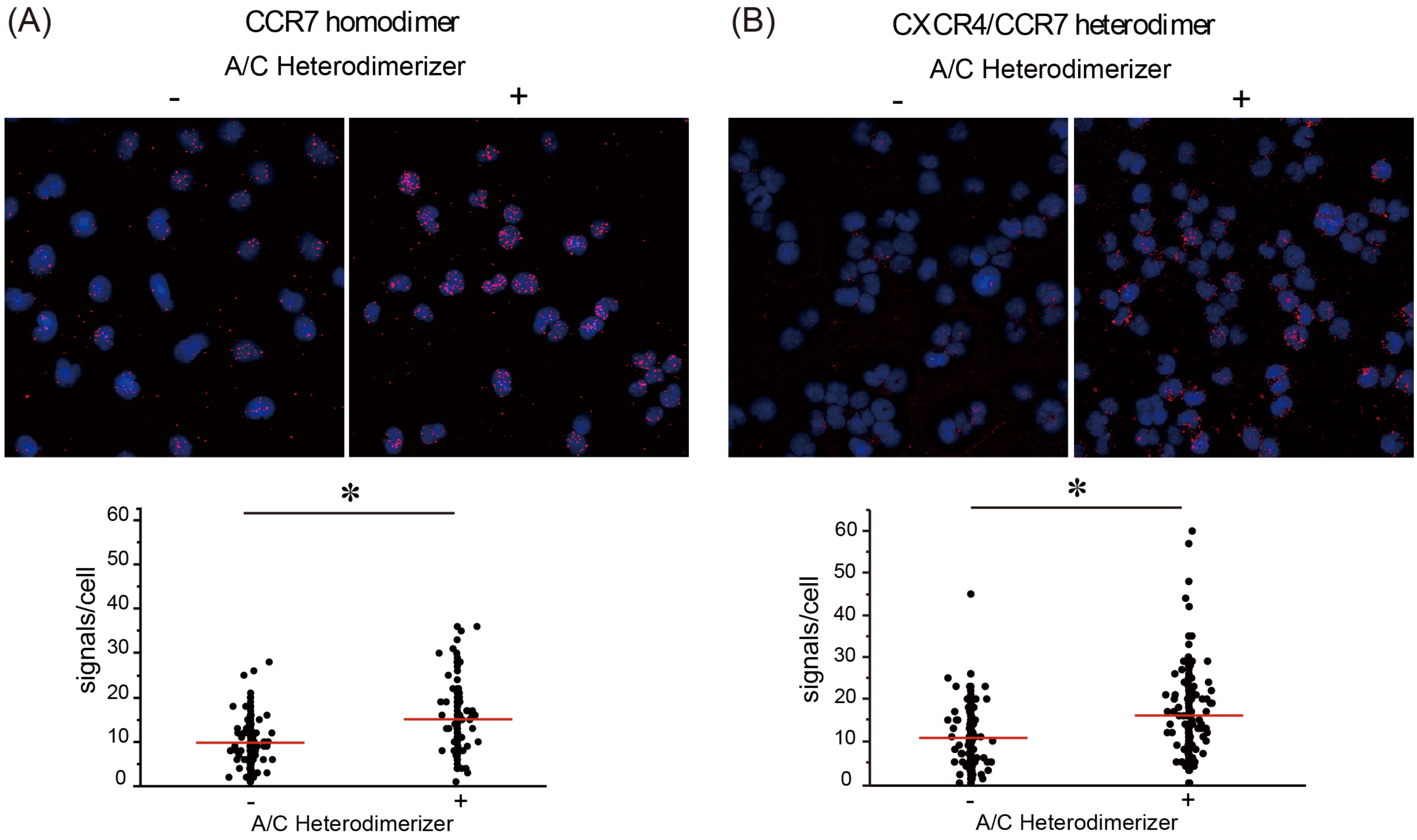

Supplemental figure 2. CCR7 homo- or hetero-dimerization is induced by A/C Heterodimerizer in the transfected H9 cell lines. (A) CCR7 homodimer formation in CCR7-DmrA/CCR7-DmrC cells after treatment with or without 500 nM A/C Heterodimerizer. The number of *in situ* proximity ligation assay (PLA) signals per cell was counted by using the Duolink Image Tool software. A representative experiment from three independent experiments is shown with the mean number of PLA signals plotted on the vertical axis. *, $p<0.05$ by Mann-Whiteny's U test.

(B) CXCR4/CCR7 heterodimer formation in CXCR4-DmrA/CCR7-DmrC cells after treatment with or without 500 nM A/C Heterodimerizer. The number of *in situ* PLA

signals per cell was counted by using the Duolink Image Tool software. A representative experiment from two independent experiments is shown with the mean number of PLA signals plotted on the vertical axis. *, $p<0.05$ by Mann-Whiteny's U test.

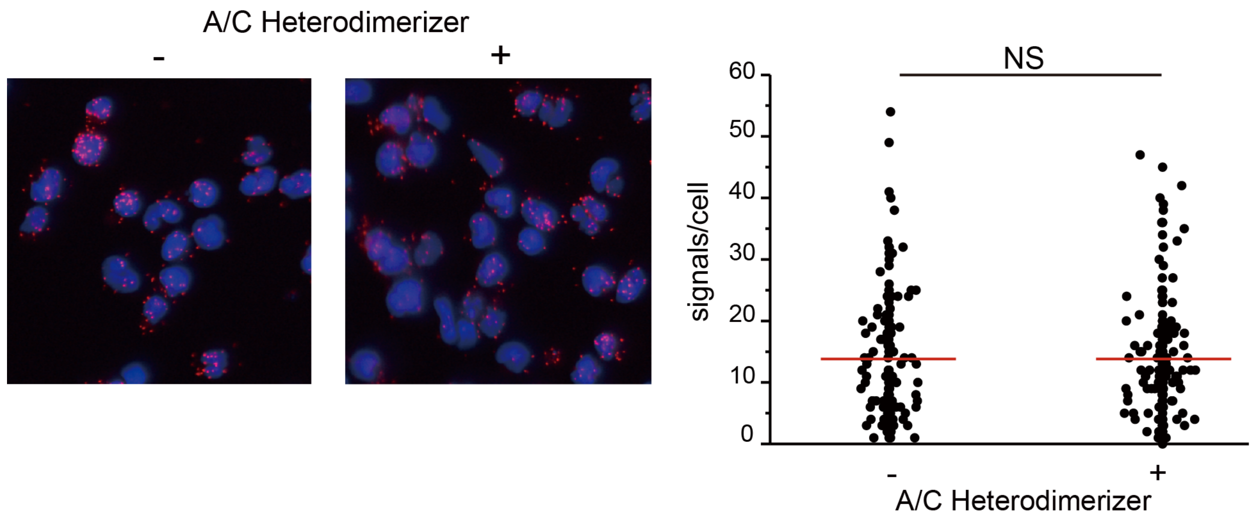

Supplemental figure 3. A/C Heterodimerizer does not affect spontaneously-formed CCR7 homodimer.

CCR7 homodimer formation after treatment with or without 500 nM A/C Heterodimerizer in H9 cells. The number of *in situ* proximity ligation assay (PLA) signals per cell was counted by using the Duolink Image Tool software. A representative experiment from three independent experiments is shown with the mean number of PLA signals plotted on the vertical axis. *, $p<0.05$ by Mann-Whiteny's U test; NS, not significant.

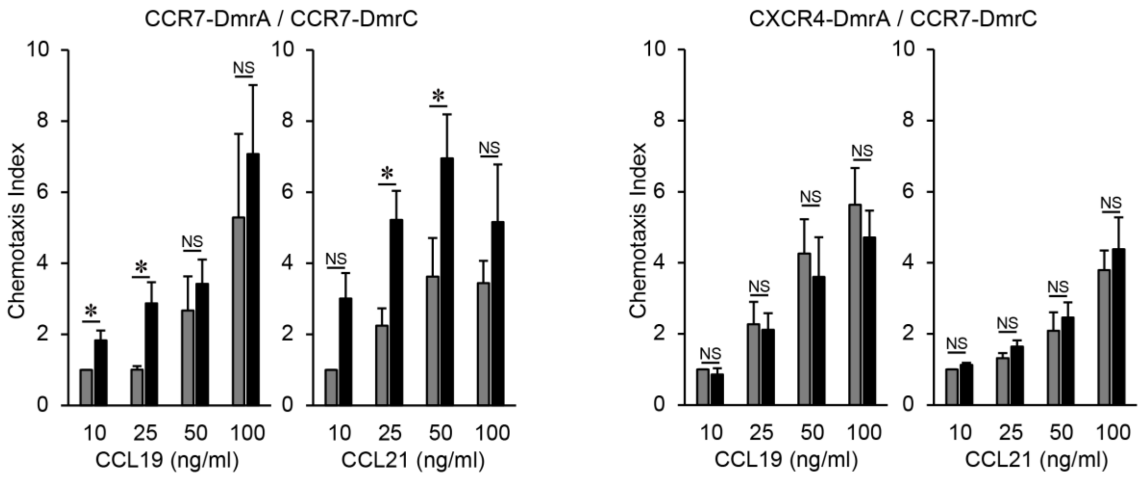

Supplemental figure 4. CCR7-dependent chemotaxis is enhanced after induction of CCR7 homodimerization.

CCL19- and CCL21-dependent chemotaxis after induction of CCR7 homo- (left) or CXCR4/CCR7 hetero (right) -dimerization were analyzed by the Transwell assay. CCL19 or CCL21 at the indicated concentration was added to the lower wells, and the stably transfected H9-derived cell lines expressing CCR7-DmrA/CCR7-DmrC (left) or CXCR4-DmrA/CCR7-DmrC (right) were added to the upper wells in the presence (black bars) or absence (gray bars) of 500 nM A/C Heterodimerizer. The relative chemotactic index shown is the ratio of the number of cells that migrated at the indicated concentration of CCL19 or CCL21 to those that migrated at 10 ng/ml CCL19 or CCL21 without A/C Heterodimerizer. Data represent mean ±SEM from three or four independent experiments. *, $p<0.05$ by Student's *t* test; NS, not significant.

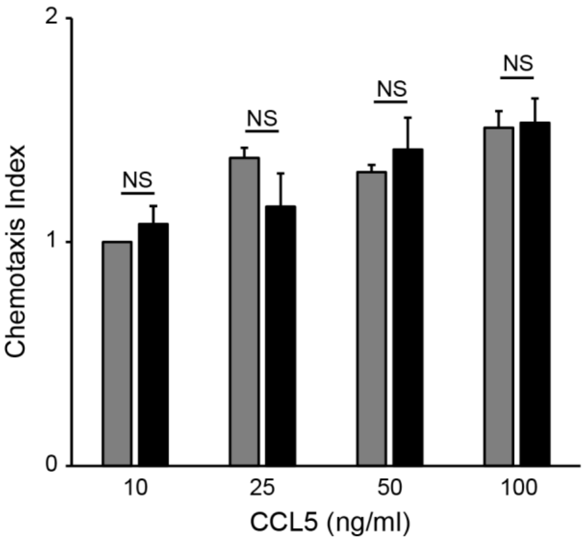

Supplemental figure 5. CCL5-dependent chemotaxis is not affected after induction of CCR7 homodimerization.

CCL5-dependent chemotaxis after induction of CCR7 homo-dimerization was analyzed by the Transwell assay. CCL5 at the indicated concentration was added to the lower wells, and CCR7-DmrA/CCR7-DmrC expressing H9 cells were added to the upper wells in the presence (black bars) or absence (gray bars) of 500 nM A/C Heterodimerizer. The relative chemotactic index shown is the ratio of the number of cells that migrated at the indicated concentration of CCL5 to those that migrated at 10 ng/ml CCL5 without A/C Heterodimerizer. Data represent mean ±SEM from four independent experiments. NS, not significant.

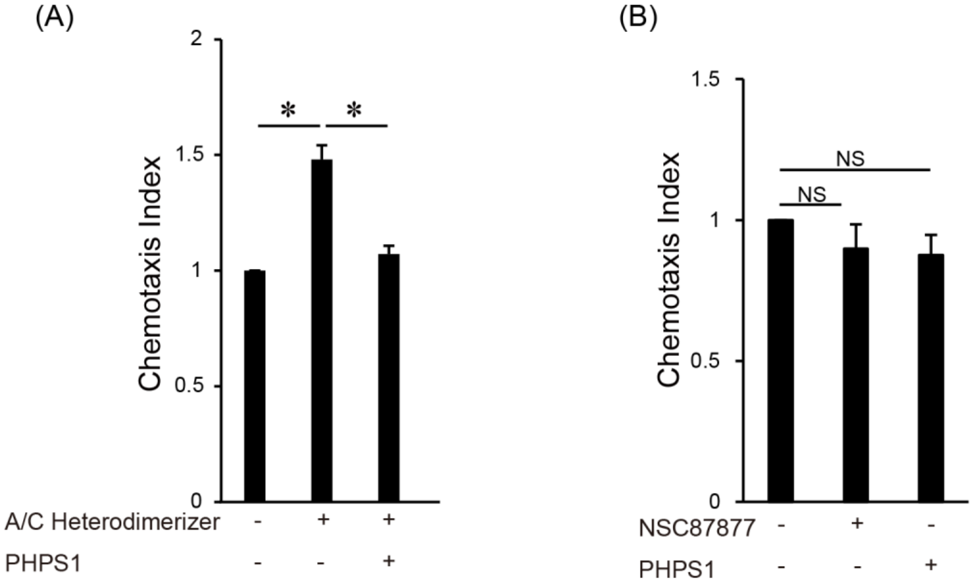

Supplemental figure 6. A SHP2 inhibitor restores the effect of CCR7 homodimerization on CCL21-induced cell migration.

(A) CCL21-dependent chemotaxis in CCR7-DmrA/CCR7-DmrC expressing H9 cells after induction of CCR7 homo-dimerization was examined with or without 5 μM PHPS1. The relative chemotactic index achieved by the number of migrated cells in response to 25 ng/ml CCL21 is shown. Data represent mean ±SEM of three independent experiments. *, $p<0.05$ by Student’s *t* test.

(B) The effect of 5 μM PHPS1 or 40 μM NSC87877 on CCL21 (25 ng/ml)-dependent chemotaxis in CCR7-DmrA/CCR7-DmrC expressing H9 cells. The relative chemotactic index in the absence of A/C Heterodimerizer is shown. Data represent mean ±SEM of five independent experiments. NS, not significant.

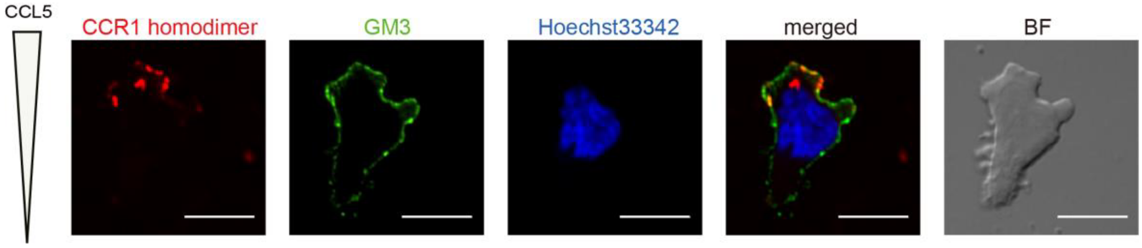

Supplemental figure 7. CCR1 homodimers are polarized toward GM3-enriched leading edge during CCR1-dependent cell migration

The localization of CCR1 homo-dimers in T cell migration in response to CCL5 is shown (Red; PLA signal, Green; anti-GM3 antibody, Blue; Hoechst 33342). H9 cells were loaded into each well of the EZ-TAXIScan microchamber. After cell alignment was completed, human CCL5 (100 ng) was applied to the contra-wells. During migration, cells were fixed and stained with anti-human CCR1 antibody conjugated with the complementary oligonucleotide probes, anti-GM3 antibody, and Hoechst 33342. Scale bar: 10 µm

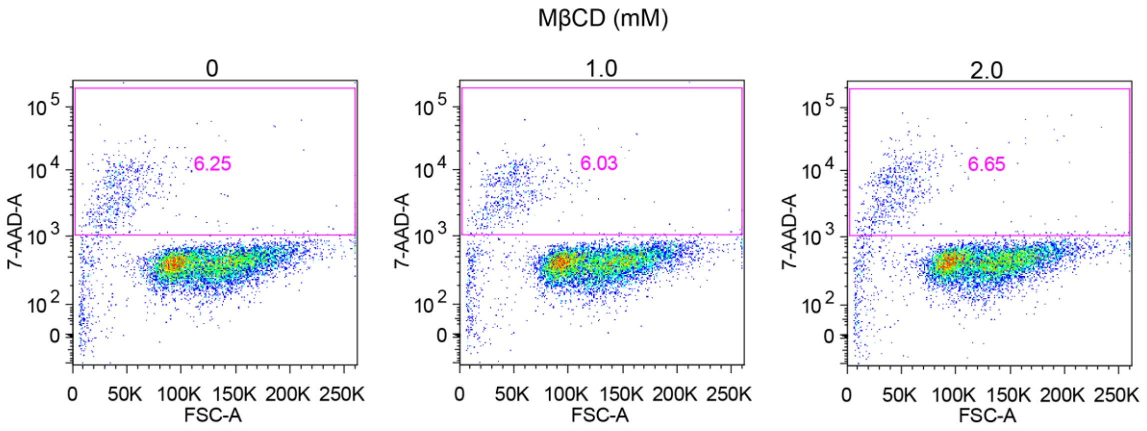

Supplemental figure 8. MβCD treatment does not affect cell viability.

H9 cells were treated with the indicated concentrations of MβCD for 30 min. The cells were resuspended and incubated in RPMI1640 medium containing 0.1% BSA for 2 hr, and then stained with 7-aminoactinomycin D (7-AAD). The samples were examined by flow cytometry, and the percentage of 7-AAD-positive dead cells is shown. A representative experiment from at least three independent experiments is shown.

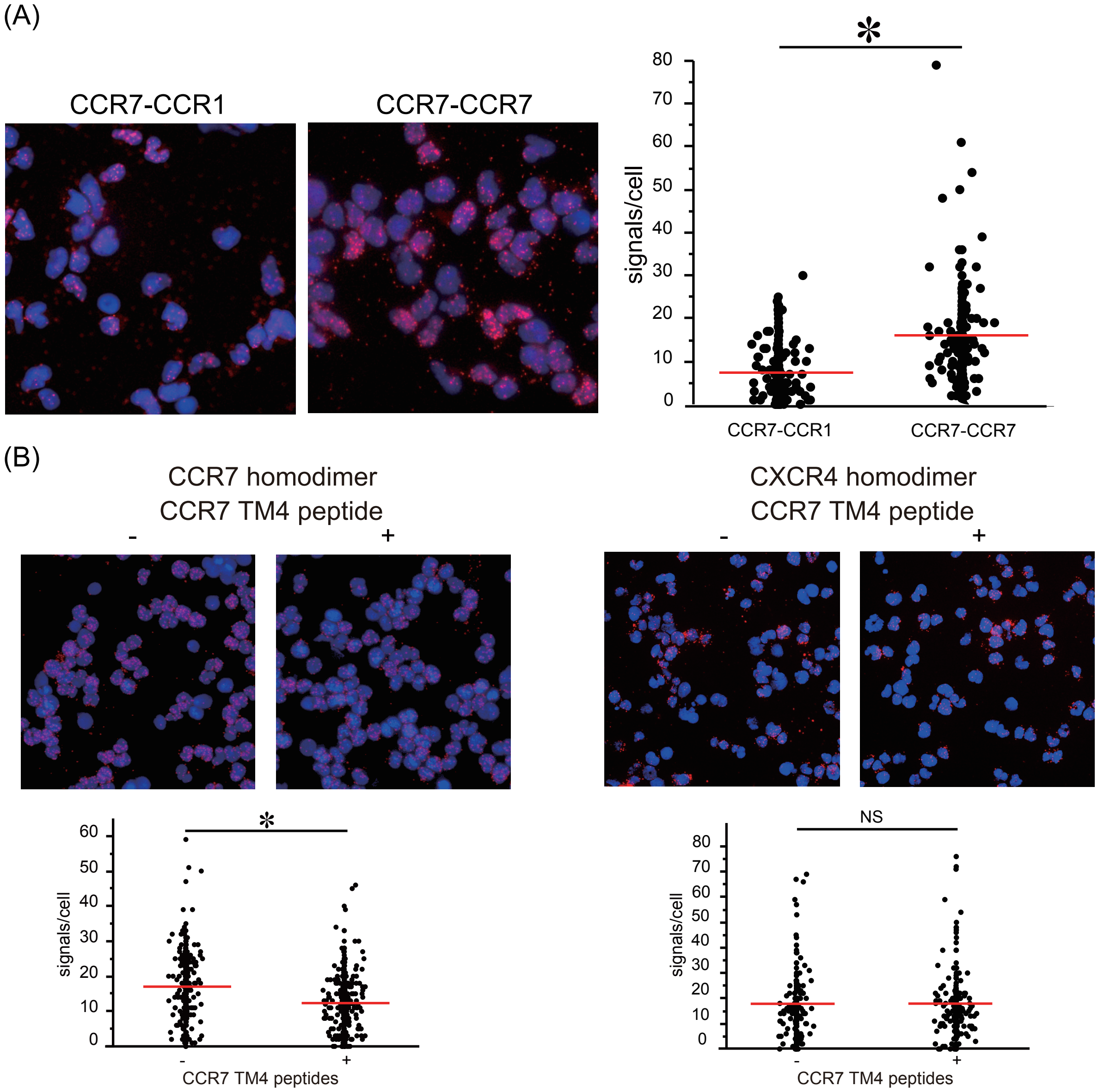

Supplemental figure 9. CXCR4 homo dimer formation is not impaired by the CCR7 TM4 peptide.

(A) CCR7/CCR1 or CCR7/CCR7-dimer formation in H9 cells was examined by *in situ* PLA. CCR7-CCR1 heterodimer formation was detected by anti-CCR7 mAb-PLA^{Minus} and anti-CCR1 mAb-PLA^{Plus} (left), and CCR7 homodimer formation by anti-CCR7 mAb-PLA^{Plus} and mAb-PLA^{Minus} (right). The number of PLA signals per cell was counted using the Duolink Image Tool software. A representative experiment from three independent experiments is shown with the mean number of PLA signals plotted on the vertical axis. *, $p<0.05$ by Mann-Whitney's U test

(B) CCR7 homodimer (left) or CXCR4 homodimer (right) formation after treatment representative experiment from three independent experiments is shown with the mean number of PLA signals plotted on the vertical axis. *, $p<0.05$ by Mann-Whitney’s U test; NS, not significant.

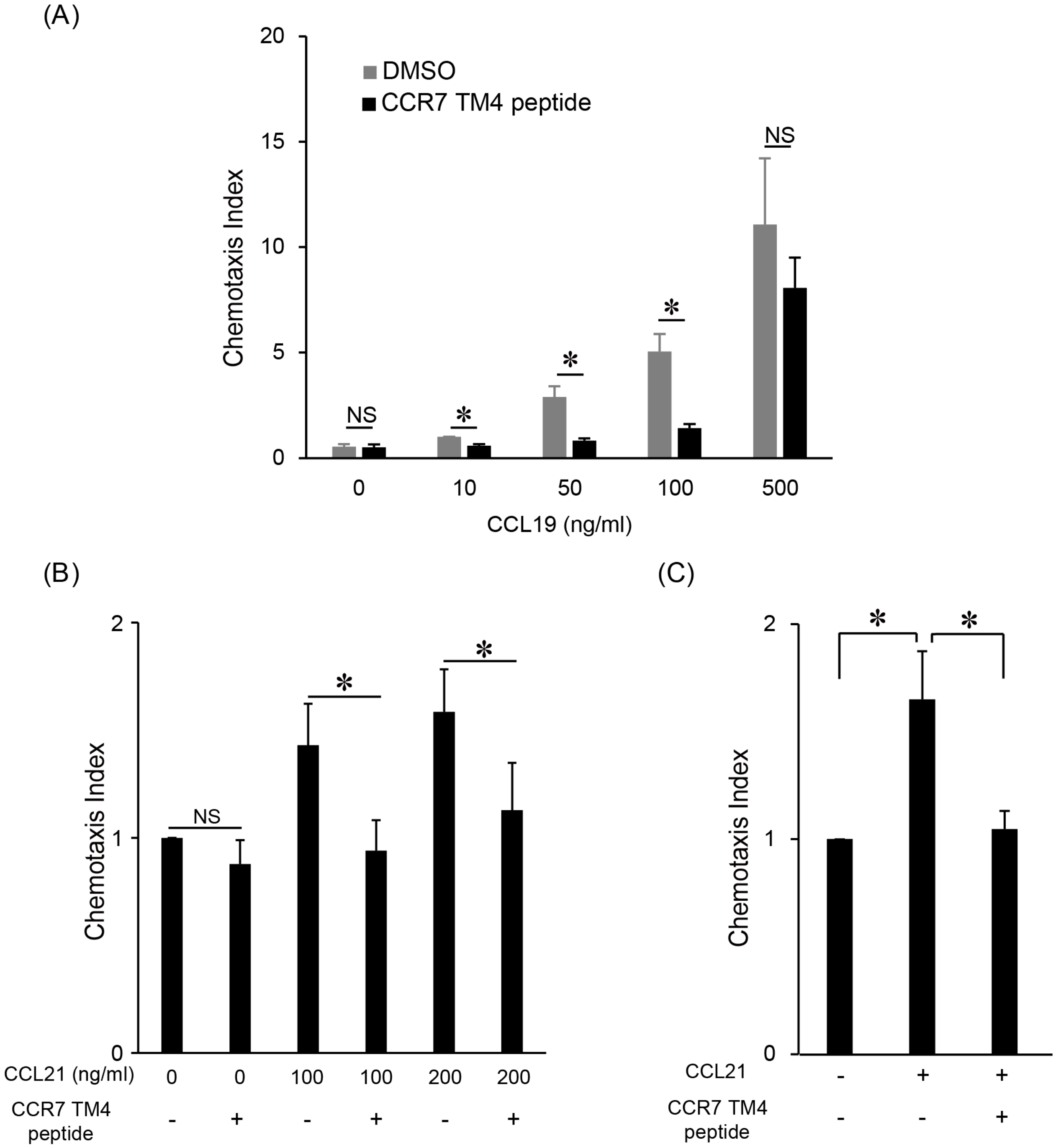

Supplemental figure 10. CCR7 ligand-induced cell migration is inhibited by the CCR7 TM4 peptide.

(A) The levels of H9 cell migration was examined with or without 15 μg/ml CCR7 TM4 peptide. The number of cells migrated in response to 0 - 500 ng/ml CCL19 were counted, and shown as a relative chemotactic index. Data represent mean ±SEM from three independent experiments. *, p<0.05 by Student's *t* test; NS, not significant.

(B) The levels of MDA231 cell migration was examined with or without 15 μg/ml CCR7 TM4 peptide. The number of cells migrated in response to 100 or 200 ng/ml CCL21 were counted, and shown as a relative chemotactic index. Data represent mean ±SEM of five independent experiments. *, $p<0.05$ by Student's *t* test.

(C) Primary human T cell chemotaxis in response to 200 ng/ml CCL21 with or without 15 μg/ml CCR7 TM4 peptide. Data represent mean ± SEM of three independent experiments. *, $p<0.05$ by Student's *t* test.

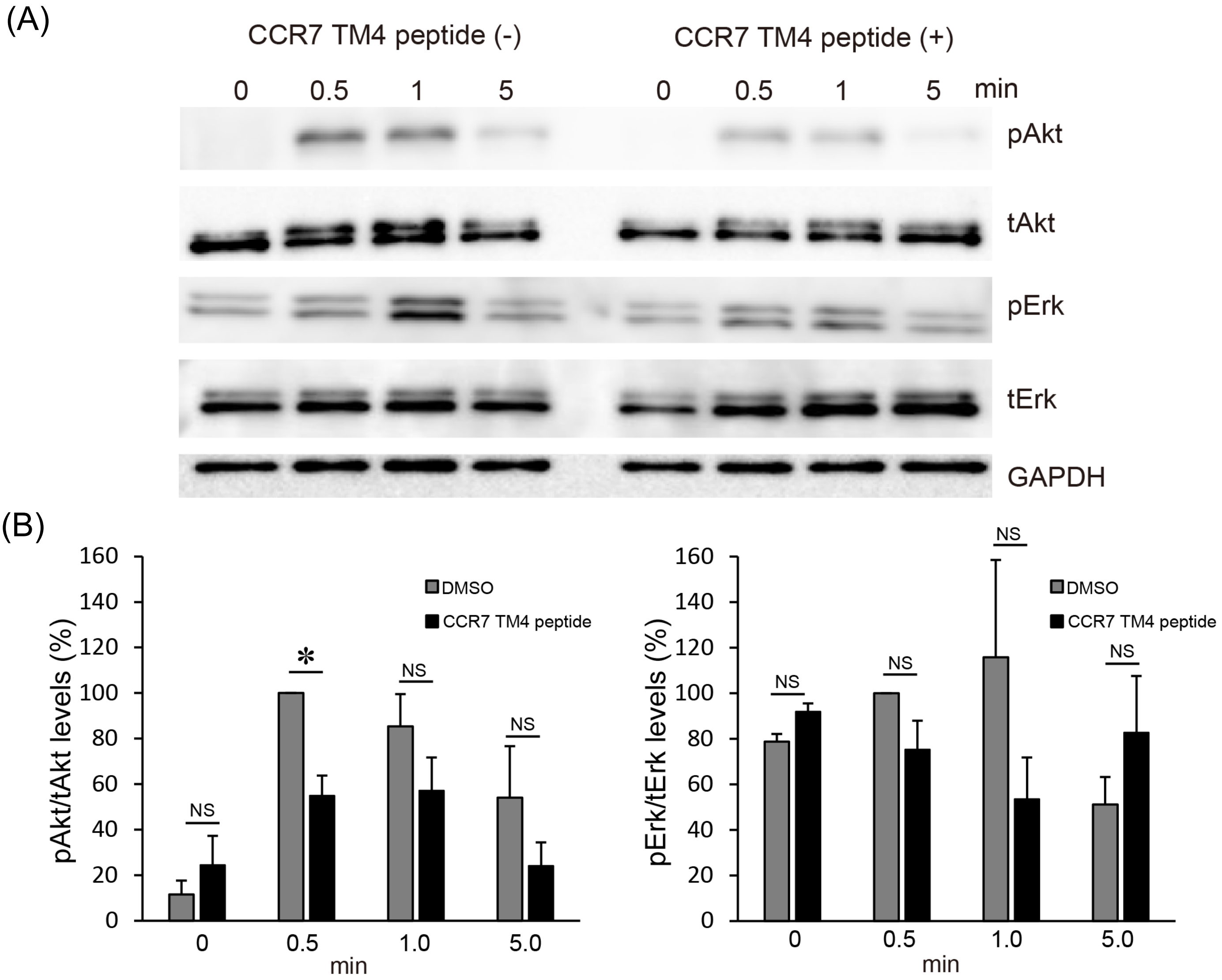

Supplemental figure 11. CCL21-induced Akt and Erk phosphorylation are inhibited by the CCR7 TM4 peptide.

(A) CCR7 ligand-induced phosphorylation of Akt and p44/42 MAPK (Erk1/2) at the indicated time points after 1 μg/ml CCL21 treatment with or without 15 μg/ml CCR7 TM4 peptide. The levels of phosphorylated and total Akt and Erk1/2 were analyzed by Western blotting using antibodies against phosphorylated Akt at Thr308 (pAkt), phosphorylated Erk1/2 (pErk), total Akt (tAkt), total Erk1/2 (tErk) or GAPDH. A representative experiment from at least three independent experiments is shown.

(B) The ratio of pAkt/tAkt (left) and pErk/tErk (right) were analyzed by densitometric

analysis. Data are expressed as percentage of pAkt or pErk levels of control treatment at 0.5 min. Data are mean ± SEM of three independent experiments. * $p<0.05$ by Student's *t* test.

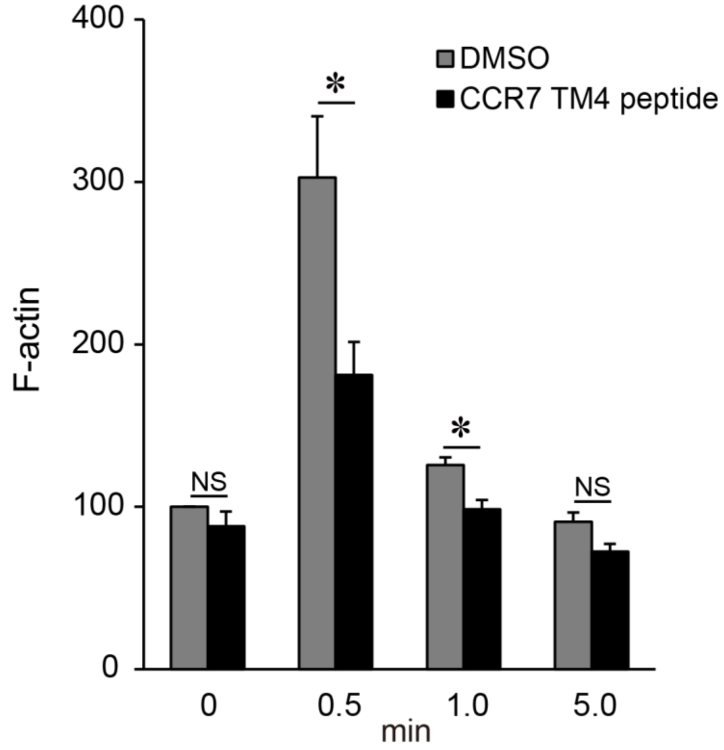

Supplemental figure 12. CCL19-induced actin rearrangement is inhibited by the CCR7 TM4 peptide.

The cells were pretreated with or without 15 μg/ml CCR7 TM4 peptide, and the F-actin levels were examined at the indicated time points after 1 μg/ml CCL19 treatment. The phalloidin staining values were analyzed by flow cytometry and are shown as MFI, where the baseline fluorescence is arbitrarily assigned a value of 100. Data are mean ± SEM of five independent experiments. *, $p<0.05$ by Student's *t* test; NS, not significant.
